# Impact of a 2-year structured program on physical fitness and physical activity in T2D patients with rapid renal function decline—the ACTIDIANE RCT

**DOI:** 10.3389/fspor.2026.1892496

**Published:** 2026-09-11

**Authors:** Laurent Bosquet, Martine Duclos, Gaël Ennequin, Elsa Heyman, Mélanie Marias, Laurent Mourot, Philippe Sosner, Oualid Ayad, Edith Bigot Corbel, Sophie Borot, Claire Carette, Bertrand Cariou, Nicolas Chevalier, Cécile Ciangura, Severine Clerjaud, Pierre-Henri Ducluzeau, Lisa Durocher, Jean François Gautier, Pierre Gourdy, Amine Kasmi, Laurence Kessler, Arnaud Monier, Pierre Morcel, Myriam Moret, Philippe Moulin, Nicolas Paquot, Stéphanie Ragot, Vincent Rigalleau, Pierre-Jean Saulnier, André Scheen, Igor Tauveron, Anne Vambergue, Tiphaine Vidal Trecan, Samy Hadjadj

**Affiliations:** 1 Laboratoire MOVE (UR20296), Université de Poitiers, Poitiers, France; 2 Service de Médecine du Sport et des Explorations Fonctionnelles, Université Clermont Auvergne, CHU de Clermont-Ferrand, Clermont-Ferrand, France; 3 Laboratoire AME2P, Université Clermont—Auvergne, Clermont Ferrand, France; 4 Univ. Lille, Univ. Artois, Univ. Littoral Côte d'Opale, ULR 7369 - URePSSS - Unité de Recherche Pluridisciplinaire Sport Santé Société, Lille, France; 5 Institut Universitaire de France, Paris, France; 6 Laboratoire Synergies, Université de Franche Comté, Besançon, France; 7 Centre de Diagnostic et de Thérapeutique & Centre d'Investigations en Médecine du Sport, Hôpital Hôtel-Dieu, Assistance Publique-Hôpitaux de Paris, Université Paris Cité, Paris, France; 8 Université de Poitiers, CHU de Poitiers, Centre d’Investigation Clinique (CIC INSERM 1402), Poitiers, France; 9 Département d’Endocrinologie (CIC INSERM 1413), Nantes Université, CHU de Nantes, Institut du Thorax, Nantes, France; 10 Service D'Endocrinologie-Diabétologie-Nutrition, Hôpital Minjoz, Université de Franche-Comté, CHU de Besançon, Besançon, France; 11 Départment de Nutrition (centre de l’obésité), Hôpital Européen Georges Pompidou, Assistance Publique-Hôpitaux de Paris (INSERM CIC1418), Université Paris Cité, Paris, France; 12 Service d’Endocrinologie, Diabétologie & Médecine de la Reproduction, Hôpital de l’Archet 2, Nice, France; 13 Département de Diabétologie, Hôpital de la Pitié-Salpêtrière, Sorbonne Université, Assistance Publique-Hôpitaux de Paris, Institut de Cardiometabolisme et Nutrition (ICAN), Paris, France; 14 Départment d’Endocrinologie-Diabetologie-Nutrition, CHU of Tours, Tours, France; 15 Université Paris Cité, Hôpital Lariboisière, Centre Universitaire du Diabète et de ses Complications, Paris, France; 16 Université de Toulouse, CHU de Toulouse (UMR1297/I2MC), Toulouse, France; 17 Département de Diabetologie, Inserm UMR 1260, Université de Strasbourg, CHU de Strasbourg, Strasbourg, France; 18 Diabétologie Endocrinologie, Centre Hospitalier Louis Pasteur, Le Coudray, France; 19 Service D'endocrinologie, de Diabétologie et des Maladies Métaboliques, Hospices Civils de Lyon, Hôpital Louis Pradel, Bron, France; 20 Nutrition et Maladies Métaboliques, Université de Liège, CHU Liège, Unité de Diabétologie, Liège, Belgique; 21 Service Endocrinologie-Nutrition, CHU Bordeaux, Hospital Haut-Lévêque, Pessac, France; 22 Service Endocrinologie Diabétologie, Université Clermont Auvergne, CHU de Clermont-Ferrand, Clermont-Ferrand, France; 23 Département Diabetes et Nutrition, Université de Lille, CHU Lille, Lille, France; 24 Immediab Lab, INSERM UMR-S1151, CNRS UMR-S8253, Institut Necker-Enfants Malades, Paris, France

**Keywords:** adherence, high-intensity physical activity, randomized controlled trial, sedentarity, type 2 diabetes

## Abstract

**Background:**

Long-term structured physical activity (PA) programs remain insufficiently evaluated in real-life settings for individuals living with type 2 diabetes (T2D). We assessed the effects of a 2-year high-intensity physical activity (HIPA) program on physical fitness and spontaneous PA.

**Methods:**

ACTIDIANE was a 21-center, open-label, randomized controlled trial comparing HIPA with standard PA recommendations (PAR) in 103 adults with T2D and rapid renal function decline. The HIPA intervention consisted of two supervised sessions per week delivered by certified adapted physical activity professionals over 24 months. Physical fitness and spontaneous PA were pre-specified secondary outcomes of the trial. Mixed models assessed group-by-time interactions over 24 months.

**Results:**

Baseline characteristics were similar between groups. Estimated $\dot{V}O_{2}\mathrm{peak}$ changed from 19.3 ± 3.1 to 22.0 ± 4.0 mL.min^−1^.kg^−1^ in the HIPA group vs. 19.9 ± 3.6 to 19.7 ± 3.7 mL.min^−1^.kg^−1^ in PAR (*p* for interaction <0.01). No group-by-time interaction was observed for sedentary time or moderate-to-vigorous PA, whether assessed by questionnaires or accelerometry. Adherence to HIPA was heterogeneous and declined over time.

**Conclusions:**

In adults with T2D and rapid renal function decline, a 2-year structured HIPA program significantly improved estimated cardiorespiratory fitness, a clinically meaningful finding given the strong association between $\dot{V}O_{2}\mathrm{peak}$ and long-term cardiovascular and all-cause mortality, but did not modify spontaneous PA or sedentary behaviours. These findings highlight both the potential physiological benefit and the feasibility challenges of implementing long-term high-intensity PA in real-life clinical settings. Strategies enhancing adherence are needed to optimize clinical impact.

## Highlights

- A 2-year supervised high-intensity physical activity program significantly improved estimated $\dot{V}O_{2}\mathrm{peak}$ (+14%) in adults with T2D and rapid renal function decline, a clinically meaningful outcome given its strong association with mortality.
- Despite the structured intervention, no group-by-time interaction was observed for moderate-to-vigorous physical activity or sedentary time, whether measured by questionnaires or accelerometry.
- Adherence was heterogeneous and declined over time, highlighting the feasibility challenges of long-term high-intensity exercise in real-life clinical settings and the need for adherence-enhancing strategies.

## 1 Introduction

Diabetes mellitus is a non-communicable disease whose prevalence is growing worldwide, particularly in urban settings (12, 21, 33). A large body of evidence has shown that regular physical activity plays a key role not only in the management but also in the prevention of type 2 diabetes (T2D), by improving glucose metabolism, body composition, and insulin sensitivity (9, 17). This disease progresses over time and can lead to severe macro- and microvascular complications, including renal failure (18). While the therapeutic arsenal has regularly improved, non-pharmacological therapies such as nutrition and physical activity (PA) remain the cornerstone of diabetes care and are consistently positioned as a first-line treatment in patients care, in international as well as national guidelines (9, 13).

The benefits of PA, and especially endurance and resistance training, have been clearly demonstrated for most pathologies (31). Focusing more specifically on individuals living with T2D, it has been clearly demonstrated that PA improved glycaemic control, notably through enhanced insulin sensitivity, glucose transport and uptake, as well as glycogenesis (19, 32). These metabolic benefits are complemented by other positive effects on body composition (25, 26) and cardiorespiratory/neuromuscular fitness (36), which contribute to decrease the risk of complication (34, 36), and improve patient quality of life (35).

The latest American Diabetes Association (ADA) and Société Francophone du Diabète (SFD) guidelines regarding T2D recommend performing at least 150 min of moderate to vigorous intensity physical activity (MVPA) and adding at least two sessions of resistance training per week (9, 13). However, the question remains open to determine which type of exercise should be prescribed. Most of the time, healthcare and PA professionals utilize moderate-intensity continuous exercises (MICE), which consist in cycling, walking or running between 20 and 60 min, at an intensity ranging from 50% to 70% of peak oxygen uptake $\left(\dot{V}O_{2}\mathrm{peak}\right)$. While this modality of exercise has proven effective for numerous therapeutic targets, including metabolic or cardiovascular outcomes (30), research over the last 15 years has clearly shown that MICE was not necessarily the most effective modality to achieve health benefits. Indeed, high-intensity interval exercise (HIIE), which consists in alternating periods of exercise lasting 30 to 300 s at an intensity ranging from 95% to 100% of $\dot{V}O_{2}\mathrm{peak}$, with recovery periods of equal, shorter, or longer duration (16), has been shown to be more effective than MICE in reducing cardiometabolic disease risk (20) and improving cardiorespiratory fitness (40). In addition to requiring less time to achieve the same energy expenditure during a session, this exercise modality is perceived as being more enjoyable than MICE, which is a major factor for long-term adherence (3).

Although HIIE can be perceived as riskier than MICE, especially from a cardiovascular viewpoint, it has become popular and is increasingly used in addition to MICE. Nevertheless, very few reports considered HIIE program on a range longer than 12 weeks, questioning its long-term safety and efficacy, particularly regarding renal complications of T2D, where evidence is lacking. The ACTIDIANE randomized controlled trial aimed to compare the safety and efficacy of a 2-year structured physical activity program using HIIE in combination with MICE vs. standard counselling for physical activity on renal function decline in people living with T2D. The impact of a physical activity program like the one proposed in ACTIDIANE has never been evaluated over such a long period (i.e., 2 years). Therefore, the aim of this analysis is to present the effect of these two interventions on pre-defined outcomes of ACTIDIANE: physical fitness, spontaneous physical activity and sedentarity.

## 2 Methods

### 2.1 Trial design

The ACTIDIANE trial is a multi-center open randomized controlled trial, conducted in two 1:1 parallel group. Its aim was to compare the effect on renal function decline of a 2-year supervised training program (High Intensity Physical Activity, HIPA) to a more conservative approach consisting in Physical Activity Recommendations, PAR that fulfilled national and international guidelines (9, 13). Once participation criteria were verified, patients were randomized in one of the two groups: PAR or HIPA. The study was designed and conducted in accordance with the Declaration of Helsinki and in compliance with the ethical principles of good clinical practice. The protocol was approved by an independent ethics committee (CPP Sud Méditerranée IV). All patients gave written informed consent before any trial-related activities.

### 2.2 Participants

ACTIDIANE participants were females and males, aged 45 years and more, with established T2D and evidence of rapid renal function decline (faster than −5 mL.min^−1^.yr^−1^). Renal function decline was assessed by creatinine-derived CKD-EPI eGFR considering all available serum creatinine determinations in a single participant in a time period of 3 to 24 months, prior to screening. Patients with baseline eGFR lower than 30 mL.min^−1^ were not included. Detailed inclusion and non-inclusion criteria are presented in Appendix A. Patients were carefully examined and a non-contraindication statement for HIIE was issued, with a special focus on cardiological risk, before randomization.

### 2.3 Tests and measures

All tests were performed locally in the hospital department. A standard operating procedure was prepared for each test to warrant reliability of the measures between the participating centres. An optional participation to an ancillary analysis was proposed to all participants regarding the determination of their body composition and the use of an accelerometer to assess PA level and sedentarity. Body composition was determined during the inclusion visit and after 12 and 24 months, while PA level and sedentarity were assessed at baseline and after 6, 12, 18 and 24 months.

#### 2.3.1 Body composition

Body composition was assessed using routine local impedancemeters, without mandatory fasting state. Participants were asked to empty their bladder prior to the test, to wear light clothes, and to remove all jewelry and metal objects.

#### 2.3.2 Physical fitness

The 10 m walk test is a functional test assessing usual gait performance. Participants walk 14 m at a usual walking velocity, and 14 m at their maximal walking velocity. Timing was recorded over the central 10 meters to exclude acceleration (2 m) and deceleration (2 m) phases. Two trials were allowed for each condition, the best one being retained for further analyses.

The 6 min step test is a submaximal exercise test evaluating cardiorespiratory fitness. Participants step up and down a step of 15 cm height at a self-paced rhythm for six minutes. Clinical research nurse/assistant used a counter to count completed steps (1 step is one up and down) during the 6 min time. Heart rate was monitored continuously using a heart rate monitor (Polar Electro Oy, Kempele, Finland). $\dot{V}O_{2}\mathrm{peak}$ was estimated from Equation 1 (24): $\;\dot{V}O_{2}\mathrm{peak}=7.849+(0.101\;\times \;\mathrm{UDSC})$, where $\dot{V}O_{2}\mathrm{peak}$ is peak oxygen uptake in mL.min^−1^.kg^−1^, and UDSC the number of up and down step cycles during the test.

#### 2.3.3 Spontaneous physical activity and sedentarity

Seven-day self-reported MVPA (in MET.h.week^−1^) and sedentarity time (h.day^−1^) were assessed during the week preceding each visit in the recruitment centre (i.e., at the inclusion, and after 6, 12, 18 and 24 months) using the International Physical Activity Questionnaire (IPAQ) (10) and the Recent Physical Activity Questionnaire (RPAQ) (14), respectively. Participants who consented to the ancillary study also worn an accelerometer (wGT3X-BT, Actigraph, USA) on the wrist of the non-dominant arm for the same 7-day periods, with continuous 24 h monitoring to obtain an objective measure of MVPA and sedentarity time (both in min.week^−1^), calculated according to the cut-points proposed by Montoye et al. (27) Accelerometer wear compliance was defined as a minimum of 10 h of wear per day over at least 4 of the 7 recording days, consistent with established recommendations for free-living monitoring.

### 2.4 Interventions

#### 2.4.1 Physical activity recommendations (PAR) group

Counseling of PA was provided, according to recommendations of the working group on Physical Activity of the French Language Diabetes Society (FLDS) (13). A specific leaflet was given during the inclusion visit to participants randomized in the PAR arm. Regular visits allowed to increase patients' compliance to the current FLDS guidelines.

#### 2.4.2 High intensity physical activity (HIPA) group

The program involved a minimum of 2 supervised exercise sessions per week for two years, representing a theoretical load of 208 sessions. Sessions lasted approximately 90 min and were implemented by a graduate supervisor with a group of 1 to 4 participants. Each session started with a 15 min warm-up and ended with a 10 min cool down. The body of the session consisted of 30 min of neuromuscular exercises, immediately followed by 30 min of cardiovascular exercises (always in the same order).

Neuromuscular exercises. Participants had to complete 3 to 4 sets of a 2 to 4-station circuit that started with exercises for maximal strength development, followed by exercises for strength endurance development. Rest period between exercises corresponded to the time required to go from one station to the other. A period of 2 to 3 min was given between circuits. If the choice of exercises was left to training centers, each of them had to fulfill American College of Sports Medicine (ACSM) guidelines for strength development in older adults (8, 32). The first session of the week had to focus on core and upper limb, while the second one had to focus on lower limb.

Cardiovascular exercises. Two exercise modalities were used to improve cardiovascular fitness: MICE or HIIE. Moderate intensity continuous exercises consisted of a single 20 to 30 min bout of exercise at a moderate intensity. High intensity intermittent exercises consisted of alternating 15 s exercise bouts at a severe intensity with 15 s of passive recovery (i.e., no exercise). Each HIIE session involved 2 sets of 12 to 14 repetitions, interspersed by a 5 min passive recovery between sets. The program included one MICE session and one HIIE session per week. The choice of the ergometer was left to the training centre, while exercise intensity was self-selected by the participants, with the help of the supervisor, based on their perceived exertion (between 12 and 14 for MICE, and between 16 and 17 for HIIE, using the Borg scale) (4).

Adaptation period. Considering the anticipated low functional capacity of most of the participants, training centres had the possibility to propose a 2- to 3-month adaptation period before starting the training program. The decision was left to the medical doctor who included the participant and/or to the graduate supervisor of the training centre. This adaptation period was intended to reassure the participants, to educate them on the participation to a structured exercise program (clothes, hydration, nutrition, warm-up, cool-down, etc) and to give them the functional capacity level required to start the training program.

Training centres. Since recruitment centres were spread and the recruitment was multicentric, a condition to implement the training intervention with included patients was to identify training centres that fulfilled a list of criteria available in Appendix B.

Adaptation to the COVID 19 pandemic. The COVID 19 pandemic started during the study and greatly impacted the implementation of the training intervention. Participants who were not able to go to their ACTIDIANE training center received a package including a camera and small equipment for exercise sessions at home (pedals, rubber bands and swiss ball). E-coaching sessions were conducted online by the supervisors, under the same conditions as the face-to-face sessions (i.e., 1 to 4 participants per session, with the same frequency and the same content). In order to take the heterogeneous digital literacy among participants into account, it has been proposed to replace videoconference sessions with teleconference sessions in some of them (t-coaching; i.e., using the telephone). Again, the same conditions as the face-to-face sessions used by the supervisors were respected. The ACTIDIANE scientific committee advised to stop patient recruitment on July 2021. This decision was validated by the Independent Data Monitoring Committee. This decision considered the insufficient pace of recruitment after the COVID-19 pandemics lockdown, potentially leading to a decreased statistical power, but also the fact that the standard deviation of eGFR slope was lower than anticipated, leading to an increased statistical power than initially planned.

### 2.5 Statistical analysis

Continuous data were presented as mean ± standard deviation (SD) or median (25th–75th percentiles) in case of skewed distribution. Numbers (exact *n* and % of the sample) were produced for qualitative variables. The evolution of each parameter was compared between the two groups using a mixed model with the interaction between group and time as fixed effect and the participant as a random effect. For all mixed models the normality assumption and the homoscedasticity of the residuals was graphically checked. If the hypotheses were not validated, we performed a square root-transformation. *P* value below 0.05 was considered as statistically significant, and the magnitude of difference between groups was assessed with Cohen's d. All the analyses were performed with R version 4.0.4.

## 3 Results

### 3.1 Baseline characteristics

**Figure 1:**
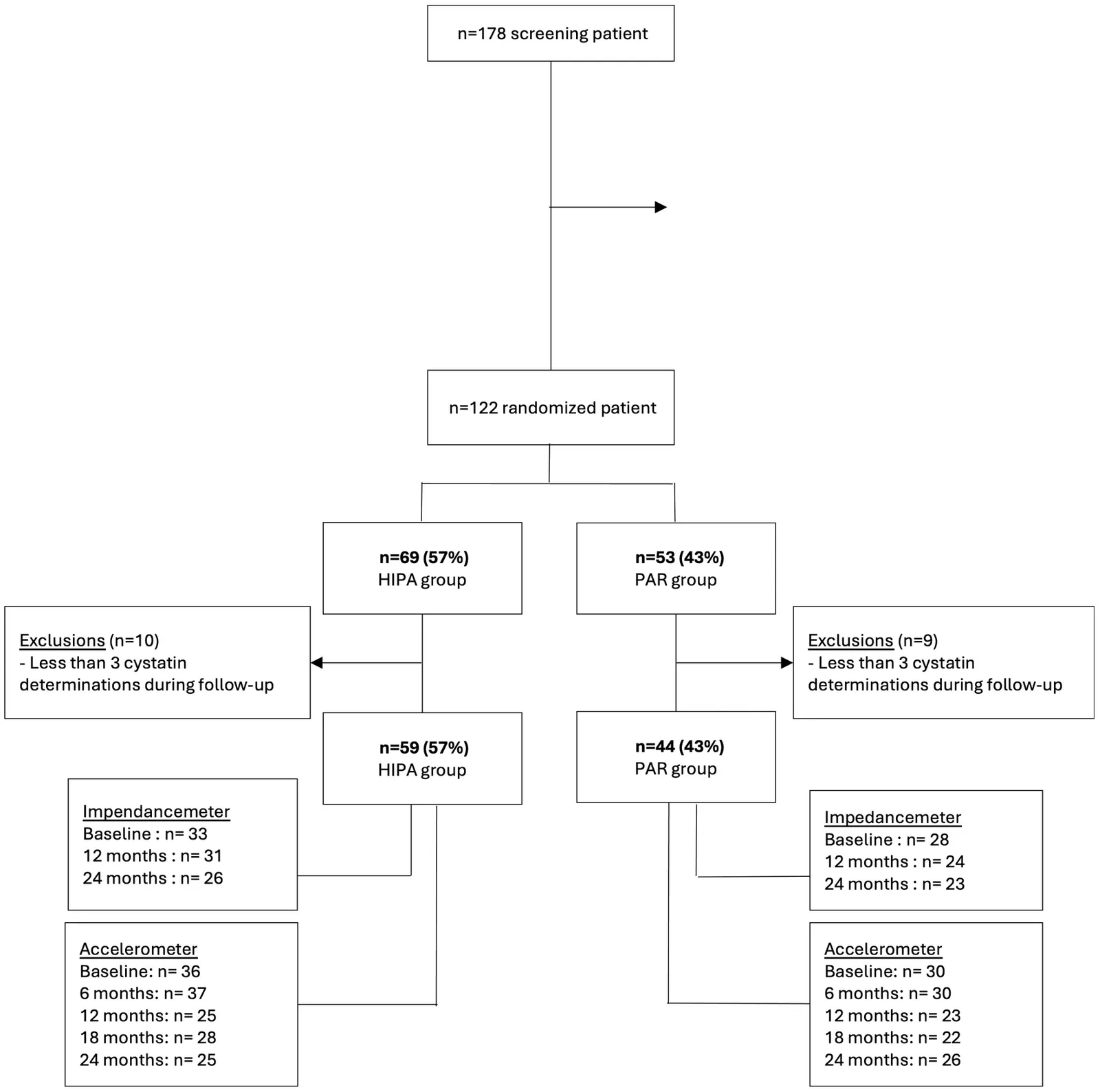
Study flowchart, with special emphasis on ancillary studies (body composition measured with an impedancemetry, or physical activity and sedentary time measured by accelerometry).

Recruitment of the participants was done in 21 hospital centres. First patient, first visit occurred on February 12th 2018 and last patient, last visit was on July 28th 2023. The study flow chart is shown in Figure 1. A total of 178 patients were screened and 122 randomized with 69 in the HIPA arm and 53 in the PAR arm. Ultimately, 103 were available for primary renal outcome analysis (59 HIPA and 44 PAR). Their baseline characteristics are presented in Table 1. We found no significant differences between both study arms except for history of heart failure (7 cases/59, 12% in HIPA and 0/44, 0% in PAR).

**Table 1:** Baseline characteristics of participants.

Variables	Overall (*n* = 103)	HIPA (*n* = 59)	PAR (*n* = 44)
Gender (Female//Male/%)	29/74 (72%)	14/45 (76%)	15/29 (66%)
Age (years)	66.1 ± 7.7	66.2 ± 8.5	65.9 ± 6.7
Body mass index (kg/m^2^)	32.9 ± 7.3	33.0 ± 7.6	32.8 ± 7.1
Body weight (kg)	93.3 ± 19.8	93.8 ± 20.4	92.7 ± 19.1
Lean body mass (kg)	59.1 ± 12.7	56.3 ± 14.3	61.4 ± 10.8
Resting HR (bpm)	72.7 ± 10.9	72.0 ± 11.0	73.6 ± 10.8
Cardiovascular disease (Yes/%)	25 (24%)	17 (29%)	8 (18%)
History of heart failure (Yes/%)	7 (7%)	7 (12%)	0 (0%)
History of myocardial infarction (Yes/%)	11 (11%)	9 (15%)	2 (5%)
History of hypertension (Yes/%)	87 (84%)	50 (85%)	37 (84%)
History of retinopathy (Yes/%)	29 (28%)	19 (32%)	10 (23%)
HbA1c (%)	7.3 (6.6–8.2)	7.4 (6.7–8.2)	7.2 (6.6–8.0)
Baseline _Creatine_ eGFR	62.9 (46.5–80.8)	62.9 (44.1–77.6)	63.0 (50.0–84.4)
Baseline _Cystatin_ eGFR	54.2 (36.8–65.1)	50.8 (35.6–61.4)	56.9 (42.7–68.3)

HIPA, high intensity physical activity; PAR, physical activity recommendations; Cardiovascular disease, coronary artery disease, myocardial infarction, stroke.

Data are presented as number (%), mean ± standard deviation or median (min–max).

### 3.2 Compliance with the HIPA program

**Figure 2:**
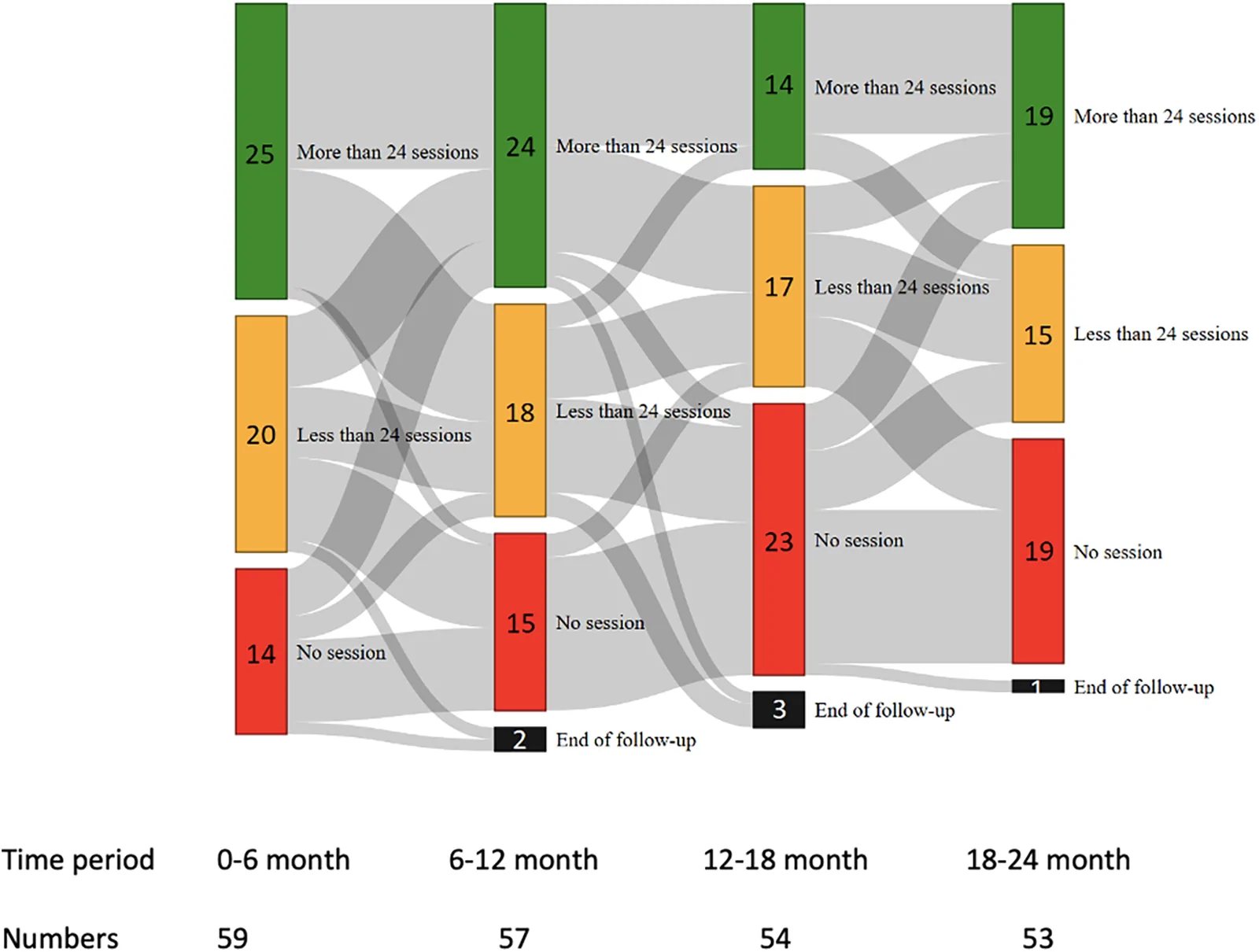
Alluvial plot of the attendance of the participants to the HIPA program.

The number of patients randomized in the HIPA group who attended 0, less than 50%, or more than 50% of the sessions, during the first, second, third and fourth semester of the study is presented in Figure 2. The time between randomization and attendance to the first HIPA session was 61 days (17–146). At the end of the 2-year program, 7 patients among the 59 participants of the HIPA group (12%) performed more than 24 sessions each semester, while 4 patients (7%) did not perform any session. Figure 2 shows the possible attendance pathways and Table 2 provides the details according to the mode of coaching (i.e., in the training center, by e-coaching or by t-coaching). T-coaching was associated to a lower training load than the two other modes of coaching.

**Table 2:** Compliance to the HIPA program of according to the semester and the mode of coaching.

Variables	Overall	tc-coaching	e-coaching	t-coaching
V2–V5 (0 to 6 months)				
Number of patients	45	38	9	0
Number of sessions,	24 (2–64)	23.5 (2–64)	24 (5–50)	/
Number of sessions per week,	0.84 (0.06–2.46)	0.86 (0.06–2.46)	0.77 (0.16–1.21)	/
Percentage of session completed,	42.0 (3.2–123.1)	43.1 (3.2–123.1)	38.4 (7.8–60.3)	/
Session duration (minutes),	64.3 ± 23.1	66.2 ± 24.6	55.5 ± 9.2	/
Training load (a.u.)	367 ± 145	372 ± 153	343 ± 91	/
V5–V6 (6 to 12 months)				
Number of patients	42	29	11	4
Number of sessions,	27 (1–60)	27 (1–58)	24 (4–60)	14 (1–26)
Number of sessions per week,	1.02 (0.03–2.23)	1.04 (0.04–2.23)	0.84 (0.22–1.50)	0.55 (0.03–1.00)
Percentage of session completed,	51.0 (1.7–111.5)	51.9 (1.9–111.5)	42.1 (11.2–75.0)	27.3 (1.7–50.0)
Session duration (minutes),	65 ± 16.7	68 ± 19	58 ± 6.3	60 ± 0
Training load (a.u.)	403 ± 147	421 ± 165	371 ± 90	319 ± 28
V6–V7 (12 to 18 months)				
Number of patients	31	21	15	3
Number of sessions,	21 (1–55)	18 (3–50)	13 (1–54)	15 (2–18)
Number of sessions per week,	0.9 (0.03–2.06)	0.62 (0.11–1.71)	0.57 (0.03–1.69)	0.59 (0.08–1.75)
Percentage of session completed,	45.1 (1.7–103.1)	31.0 (5.6–85.6)	28.3 (1.7–84.4)	29.3 (4.0–87.5)
Session duration (minutes),	68.2 ± 20.1	75.1 ± 23.2	57.7 ± 5.8	60 ± 0
Training load (a.u.)	396 ± 142	420 ± 171	364 ± 71	334 ± 30
V7–V8 (18 to 24 months)				
Number of patients	34	17	16	6
Number of sessions,	25 (1–67)	18 (1–51)	28 (1–67)	15 (12–25)
Number of sessions per week,	1.02 (0.04–2.21)	0.75 (0.04–1.96)	1.09 (0.04–2.18)	0.79 (0.37–1.09)
Percentage of session completed,	50.9 (2.2–110.7)	37.5 (1.8–98.1)	54.5 (2.2–108.9)	39.4 (18.4–54.5)
Session duration (minutes),	60.8 ± 14.7	66.1 ± 20.7	57.3 ± 9.2	60 ± 0
Training load (a.u.)	378 ± 137	428 ± 195	356 ± 79	323 ± 49

HIPA, high intensity physical activity; tc-coaching, training center coaching; e-coaching, online coaching; t-coaching, telephone coaching; training load was assessed by multiplying session duration by the perceived exertion using the s-RPE scale (Borg, 1998). Of note, numbers presented here considered only those attending at least one HIPA session during the corresponding semester.

Data are presented as numbers, numbers (min–max) or mean ± standard deviation. The number of sessions per week and percentage of session completed are reported as median (min – max).

### 3.3 Effect on sedentarity and physical activity

Subjective (by questionnaire) and objective (by accelerometry) measures of sedentary time and MVPA are presented in Table 3. There was no difference between groups at inclusion. We did not observe any interaction between groups along the 2-year intervention, regardless of the variable or the measurement method (0.093 < *p* for interaction <0.673). It is likely that the small sample size was responsible for this absence of difference between groups since statistical power was less than 80%.

**Table 3:** Impact of the ACTIDIANE program on sedentary time and moderate to vigorous physical activity, according to randomization arm.

Variables	HIPA (*n* = 59)	PAR (*n* = 44)	Cohen's d (95% CI)	*p*-value for interaction
Questionnaires				
Moderate to Vigorous Physical Activity (in min.week^−1^)				0.095
Baseline (*n* = 56//43)	182 (30–480)	120 (45–390)	0.04 (−0.36 to 0.44)	
6-month visit (*n* = 57//43)	240 (120–480)	168 (50–420)	0.15 (−0.24 to 0.55)	
12-months visit (*n* = 55//39)	180 (40–540)	240 (120–720)	−0.01 (−0.42 to 0.41)	
18-months visit (*n* = 47//38)	180 (55–380)	240 (55–600)	−0.55 (−1.00 to −0.11)	
24-months visit (*n* = 52//39)	180 (15–390)	290 (20–420)	−0.21 (−0.63 to 0.21	
Moderate to Vigorous Physical Activity (in mets.h.week^−1^)				0.093
Baseline (*n* = 56//43)	14.8 (2.6–36.7)	14.0 (3.7–38.5)	0.07 (−0.33 to 0.46)	
6-month visit (*n* = 57//43)	22.0 (10.0–41.0)	14.2 (4.0–28.0)	0.22 (−0.18 to 0.62)	
12-months visit (*n* = 55//39)	17.3 (3.7–42.2)	22.0 (8.0–58.0)	0.05 (−0.36 to 0.46)	
18-months visit (*n* = 47//38)	16.5 (4.7–35.0)	18.0 (4.0–46.8)	−0.50 (−0.94 to −0.06)	
24-months visit (*n* = 52//39)	14.0 (1.0–30.4)	20.4 (1.3–40.0)	−0.22 (−0.63 to 0.20)	
Sedentary time (h.day^−1^)				0.673
Baseline (*n* = 56//43)	4.7 (3.7–6.3)	5.1 (4.4–6.5)	−0.22 (−0.62 to 0.18)	
6-month visit (*n* = 57//43)	4.5 (3.0–5.7)	5.3 (3.9–7.1)	−0.34 (−0.74 to 0.07)	
12-months visit (*n* = 55//39)	4.4 (2.6–6.5)	4.5 (3.5–6.6)	−0.21 (−0.63 to 0.20)	
18-months visit (*n* = 47//38)	4.8 (3.1–6.5)	4.9 (4.0–6.5)	−0.23 (−0.66 to 0.20)	
24-months visit (*n* = 50//39)	4.2 (3.0–6.2)	5.0 (4.0–6.1)	−0.10 (−0.52 to 0.32)	
Accelerometer				
Moderate to Vigorous Physical Activity (in h.day^−1^)				0.442
Baseline (*n* = 36//29)	1.9 (1.4–2.7)	2.0 (1.4–2.6)	0.09 (−0.41 to 0.57)	
6-month visit (*n* = 36//28)	2.2 (1.6–3.0)	1.8 (1.4–2.4)	0.45 (−0.06 to 0.96)	
12-months visit (*n* = 25//23)	2.1 (1.3–2.7)	1.9 (1.0–2.6)	0.21 (−0.36 to 0.78)	
18-months visit (*n* = 27//22)	2.1 (1.3–2.5)	1.9 (1.7–2.6)	0.08 (−0.48 to 0.64)	
24-months visit (*n* = 25//26)	2.1 (1.3–2.5)	1.9 (1.7–2.6)	−0.07 (−0.62 to 0.48)	
Sedentary time (in h.day^−1^)				0.425
Baseline (*n* = 36//29)	8.8 (7.7–10.2)	9.0 (7.8–10.3)	−0.02 (−0.51 to 0.46)	
6-month visit (*n* = 36//28)	8.5 (7.6–10.2)	9.8 (8.2–11.3)	−0.51 (−1.02 to 0.00)	
12-months visit (*n* = 25//23)	9.1 (8.0–10.3)	9.3 (8.4–11.9)	−0.19 (−0.76 to 0.38)	
18-months visit (*n* = 27//22)	8.8 (7.9–10.5)	8.6 (8.1–9.9)	0.03 (−0.54 to 0.59)	
24-months visit (*n* = 25//26)	8.9 (8.0–11.2)	9.0 (8.0–10.1)	0.07 (−0.48 to 0.62)	

CI, confidence interval.

Data are presented as median (25th and 75th percentile). The number of patients with available data in each group is presented for each measure (*n* = HIPA//PAR).

### 3.4 Effect on physical fitness

Measures collected during the 6 min step test and the 10 m walk test are presented in Table 4. Again, we found no difference between groups at inclusion. Regarding the 6 min step test, we observed an interaction between time and group (*p* < 0.01). Participants in the HIPA group improved the number of steps and estimated $\dot{V}O_{2}\mathrm{peak}$, while these variables did not change in the PAR group. However, there was no effect of time or interaction between groups for peak heart rate. To verify that the observed imbalance regarding heart failure history (7 cases in the HIPA group vs. 0 in the PAR group) did not affect patients' ability to perform the exercise tests and, consequently, the study results, a sensitivity analysis excluding these 7 patients was performed. The results of this analysis were comparable to those obtained in the main analysis, indicating that this imbalance did not have a significant effect on our conclusions. In addition, we did not observe any effect of time or interaction between groups during the 10 m walk test, regardless of whether it was spontaneous walking speed or maximal walking speed. Again, the lack of statistical power for this secondary outcome makes it difficult to conclude to an absence of difference between groups.

**Table 4:** Impact of the ACTIDIANE program on performance during the 6 min step test and the 10 m walk test, according to randomization arm.

Variables	HIPA (*n* = 59)	PAR (*n* = 44)	Cohen's d (95% CI)	*p*-value for interaction
6 min step test				
Number of steps (*n*)				0.004
Baseline (*n* = 58//42)	113.8 ± 30.6	119.4 ± 35.8	−0.17 (−0.57 to 0.23)	
6-month visit (*n* = 57//37)	131.3 ± 40.9	125.0 ± 36.0	0.16 (−0.25 to 0.58)	
12-months visit (*n* = 46//36)	141.4 ± 35.4	120.3 ± 39.2	0.58 (0.12 to 1.03)	
18-months visit (*n* = 35//30)	129.9 ± 44.8	116.1 ± 34.7	0.35 (−0.15 to 0.84)	
24-months visit (*n* = 39//30)	140.1 ± 40.0	117.4 ± 36.5	0.59 (0.09 to 1.09)	
Peak heart rate (bpm)				0.200
Baseline (*n* = 57//38)	113.3 ± 23.8	116.3 ± 22.4	−0.13 (−0.54 to 0.28)	
6-month visit (*n* = 54//37)	113.1 ± 25.7	115.7 ± 18.0	−0.11 (−0.53 to 0.31)	
12-months visit (*n* = 45//35)	118.7 ± 23.7	111.5 ± 17.4	0.34 (−0.11 to 0.79)	
18-months visit (*n* = 35//29)	114.7 ± 24.6	111.0 ± 18.6	0.17 (−0.32 to 0.66)	
24-months visit (*n* = 39//29)	116.9 ± 23.4	112.8 ± 16.2	0.20 (−0.28 to 0.68)	
Estimated peak oxygen uptake (mL.min^−1^.kg^−1^)				0.004
Baseline (*n* = 58//42)	19.3 ± 3.1	19.9 ± 3.6	−0.17 (−0.57 to 0.23)	
6-month visit (*n* = 57//37)	21.1 ± 4.1	20.5 ± 3.6	0.16 (−0.25 to 0.58)	
12-months visit (*n* = 46//36)	22.1 ± 3.6	20.0 ± 4.0	0.58 (0.12 to 1.03)	
18-months visit (*n* = 35//30)	21.0 ± 4.5	19.6 ± 3.5	0.35 (−0.15 to 0.84)	
24-months visit (*n* = 39//30)	22.0 ± 4.0	19.7 ± 3.7	0.60 (0.09 to 1.09)	
10 m walk test				
Spontaneous walking speed (m.sec^−1^)				0.241
Baseline (*n* = 59//44)	1.16 ± 0.23	1.14 ± 0.31	0.07 (−0.32 to 0.46)	
12-months visit (*n* = 51//38)	1.19 ± 0.24	1.10 ± 0.26	0.35 (−0.08 to 0.78)	
24-months visit (*n* = 46//35)	1.17 ± 0.24	1.08 ± 0.21	0.39 (−0.06 to 0.84)	
Maximal walking speed (m.sec^−1^)				0.510
Baseline (*n* = 59//44)	1.64 ± 0.34	1.53 ± 0.42	0.30 (−0.1 to 0.69)	
12-months visit (*n* = 51//37)	1.62 ± 0.35	1.51 ± 0.33	0.34 (−0.1 to 0.76)	
24-months visit (*n* = 45//35)	1.67 ± 0.33	1.46 ± 0.33	0.64 (0.17 to 1.1)	

CI, confidence interval.

Data are presented as mean ± standard deviation. The number of patients with available data in each group is presented for each measure (*n* = HIPA//PAR).

## 4 Discussion

To the best of our knowledge, the ACTIDIANE randomized controlled trial is the first to evaluate the efficacy and feasibility of a 2-year structured high-intensity physical activity program implemented by certified professionals outside the laboratory, in real-life conditions among patients living with T2D. The main findings indicate that, despite the overall low adherence to the prescribed program, participants in the HIIE group demonstrated an improvement in estimated $\dot{V}O_{2}\mathrm{peak}$ compared with the PAR group, while no differences were observed in sedentary time or MVPA. Beyond its efficacy, this study also aimed to assess the feasibility of a medically prescribed physical activity program among individuals living with T2D and rapid renal function decline, and across heterogeneous environmental contexts (rural, urban, and peri-urban settings with varying access to facilities and qualified professionals). The present discussion therefore addresses three main aspects: (1) the feasibility and adherence patterns observed throughout the intervention; (2) the effects of the program on objectively measured physical activity and sedentary behaviours; and (3) the improvements in physical fitness, with particular attention to estimated $\dot{V}O_{2}\mathrm{peak}$ and walking performance.

### 4.1 Compliance to the program

This program was free for participants and was organized in a way that minimized travel time from home (accredited fitness centre when available, home-based coaching when there was no accredited fitness centre in the immediate neighbourhood, and occasionally e-coaching or t-coaching during the COVID-19 pandemics). As shown in Figure 2, compliance to the program is heterogeneous and not linear over the 2-year period. There are many trajectories, but the majority tends toward a progressive increase in participation during the first year of the program, followed by a stabilization or a progressive decrease during the second year. This observation is not new, as programs lasting more than six months generally show a decline in motivation over time (11). Cadmus et al. (6) examined the demographic, psychosocial, and physiological predictors of adherence to a one-year physical activity program among inactive but healthy adults aged 40 to 75. Physical activity increased during the first six months, and gradually declined over the following six months to return to baseline level. Among the predictors of adherence were program duration, body mass index, pain, and social support. This role of social support was confirmed in the randomized controlled trial by Cox et al. The authors compared two approaches to deliver a 6- to 12-month home-based PA program in inactive individuals at risk of cognitive decline, with a mean age of 70 ± 6 years old: goal-setting with mentor support or education and peer contact. Adherence to both programs remained high throughout the protocol, a finding the authors largely attributed to social support, whether from peers or mentors. It is clear from this study that the ACTIDIANE program brings together several factors that may explain its low adherence. Beyond its duration (two years), which made it vulnerable to a progressive decline in adherence (6, 11), the ACTIDIANE program was also impacted by the COVID-19 pandemics. Solutions were proposed to help participants continue the program, notably through e-coaching or t-coaching, but social isolation remained significant and inevitably influenced adherence. This was especially true for patients whose only option was t-coaching.

### 4.2 Physical activity and sedentarity

The absence of interaction between groups regarding MVPA and sedentary time contrasts with the literature, notably with the trial Lifestyle Interventions and Independence for Elders (LIFE) (15). This multicenter randomized controlled trial included 1,635 inactive adults aged 70 to 89 years with functional limitations and evaluated the impact of a 24-month structured PA program on MVPA. The intervention consisted of at least two supervised sessions per week, combining walking, strength training, flexibility, and balance exercises. When assessed by self-reported questionnaires, the LIFE study reported a significant group-by-time interaction, with the experimental group showing a greater increase in MVPA than the control group over the 24-month period (29). MVPA was still higher in the intervention group when it was assessed objectively by accelerometry, but there was no interaction between groups (Pahor et al. 29). These findings suggest that a structured, progressive and supervised program that is close to the ACTIDIANE program can lead to improvements in MVPA among older adults. Several contextual and methodological factors may explain the absence of a similar effect in our study. First, a substantial part of the ACTIDIANE program took place during the COVID-19 pandemic, a period marked by significant restrictions that limited opportunities for physical activity while promoting sedentary behaviors. In addition, the considerable heterogeneity observed in adherence levels across participants (Figure 2 and Table 2) made interpretation of the results more complex. In fact, the diversity of individual trajectories did not allow us to identify sufficiently homogeneous profiles to allow for meaningful subgroup analyses. It is generally acknowledged that a proportional relationship exists between adherence to a physical activity program and improvements in physical activity behaviors. This is illustrated by the randomized controlled trial by Mukherji et al. (28), in which 357 adults with T2D were randomized to usual care or to a structured PA program delivered either once or three times per week for a period of 6 months, in accordance with ADA guidelines. Improvements in PA and glycemic control (HbA1c) were observed specifically in the group assigned to three weekly sessions and who attended at least 50% of the prescribed exercise sessions. Our findings underscore the difficulty of translating directly conclusions from controlled trials conducted in experimental settings and over relatively short durations to real-life settings and long-duration programs such as ACTIDIANE, where implementation conditions and adherence patterns are far more variable. The heterogeneity in adherence also raises the question of whether dose-response analyses could help clarify the relationship between exposure to the HIPA program and changes in spontaneous PA or cardiorespiratory fitness. While such analyses were not pre-specified in the original protocol, the diversity of individual trajectories and the absence of a clearly defined threshold for an adequate dose precluded reliable *post-hoc* subgroup definitions. Future trials implementing long-term exercise interventions in real-life settings should prospectively define adherence thresholds and plan per-protocol sensitivity analyses accordingly.

Unlike many trials on PA, the ACTIDIANE participants in the control group (PAR) received leaflets encouraging changes in their habitual PA and were repeatedly asked about their usual PA levels, implicitly reinforcing the idea that their engagement was monitored and valued. Findings from the Italian Diabetes and Exercise Study 2 (IDES-2) (2) support this idea. In this trial, 300 physically inactive and sedentary adults with T2D were randomized to either an intervention group receiving annual theoretical and practical counselling — an approach closely resembling the support provided to our PAR group — or to a control group receiving standard care. The authors observed an improvement in MVPA in both groups, consistent with the notion that even minimal but structured encouragement can lead to positive changes in PA behaviour. A similar tendency was observed in our trial for the PAR group. However, one of the unexpected consequences was that questionnaire-based MVPA tended to increase slightly more in the PAR group than in HIPA, which may suggest that participants receiving supervised training perceived the structured sessions as sufficient for their health and therefore felt less need to further increase spontaneous activity outside the program. Such compensatory reductions in PA among individuals undertaking structured training interventions have been reported previously (26). However, it is uncertain whether this occurred in our study, since this trend toward an interaction was not observed when PA was assessed objectively by accelerometry, indicating a possible tendency for the PAR group to overestimate their actual MVPA, as previously reported for the IPAQ (39), and for other self-report questionnaires (38).

### 4.3 Physical fitness

We observed an interaction between time and group for estimated $\dot{V}O_{2}\mathrm{peak}$, with a 14% increase at 24 months in the HIPA group, compared to a 1% decrease in the PAR group. Considering that $\dot{V}O_{2}\mathrm{peak}$ is a strong predictor of mortality (22), an increase such as observed in our study is likely to contribute to better long-term health outcomes in individuals with T2D. This effect is in accordance with findings from previous meta-analyses. Boulé et al. (5) analyzed 8 randomized controlled trials involving 266 adults with T2D and a mean age of 56 years old, and that evaluated the impact of structured physical exercise interventions lasting at least 8 weeks on cardiorespiratory fitness. They reported a mean improvement in $\dot{V}O_{2}\mathrm{peak}$ of 11.8% compared to control conditions. More recently, the meta-analysis by Al-Mhanna et al. (1) included 20 studies focusing specifically on individuals with T2D and overweight or obesity. The authors reported a small but consistent improvement in $\dot{V}O_{2}\mathrm{peak}$ across interventions, reinforcing the notion that even modest increases in aerobic fitness can be expected with long-term structured physical activity programs in this population. Considering that $\dot{V}O_{2}\mathrm{peak}$ was estimated from the number of steps completed during the 6 min step test, the observed improvement may be the consequence of an enhancement in cardiorespiratory fitness, an enhancement in neuromuscular fitness, or both. Data available in our study do not allow to clearly determine the underlying mechanisms of this improvement whether they are central (e.g., increased cardiac output resulting from enhanced stroke volume, as heart rate remained unchanged) or peripheral (e.g., improved arteriovenous oxygen difference or neuromuscular efficiency). However, Chinnappa et al. (7) showed in patients with chronic kidney disease that arteriovenous oxygen difference was a stronger predictor of $\dot{V}O_{2}\mathrm{peak}$ than cardiac output, thereby highlighting the importance of peripheral adaptations in this population.

The 10 m walk test was included as a complementary functional measure to capture short-distance gait performance. The average velocity in this test is a valid and reliable marker of mobility and is strongly associated with functional independence (37). Given the high prevalence of peripheral neuropathy, muscle weakness and sarcopenia in individuals with T2D and renal complications (23), this test was expected to provide clinically relevant information not fully reflected by $\dot{V}O_{2}\mathrm{peak}$ estimates. We did not find a group-by-time interaction for either spontaneous or maximal walking speed. Evidence on the sensitivity of short-distance walking speed to structured physical activity programs in T2D with renal impairment remains scarce, particularly over long durations such as in ACTIDIANE. Comparisons with older adult populations—where walking speed improvements are often observed following multimodal training—should be made cautiously, as baseline functional status and adaptive potential differ substantially. The relatively high baseline maximal walking speed in our sample may also have limited the margin for improvement. Furthermore, heterogeneity in adherence likely increased variability and reduced the ability to detect subtle changes in gait speed. Finally, it cannot be excluded that diabetes-induced renal decline itself substantially reduced the adaptive capacity of skeletal muscle. Bittel et al. (2021) reported that the progression of diabetic kidney disease is associated with downregulation of transcriptional networks controlling oxidative phosphorylation, impairments in mitochondrial coupling efficiency, and corresponding reductions in muscle performance and physical function. Whether such mitochondrial alterations are reversible or modifiable through long-term structured exercise remains uncertain, but they may have contributed to the limited response observed in our population.

### 4.4 Limitations and future directions

This study presents several limitations that should be considered when interpreting the findings. First, physical activity and sedentary time were objectively measured using wrist-worn accelerometers, a method known to overestimate physical activity levels, particularly during non-ambulatory upper body movements. However, as our objective was to examine within-individual changes over time, rather than to quantify absolute levels, the relative validity of the device remains appropriate for assessing intervention effects. Furthermore, the use of wrist placement ensured better wear compliance over the long follow-up period, which was critical for minimizing missing data.

Second, the sample size obtained was lower than originally expected, because 120 patients were initially required in each group for evaluating renal outcome. However, the study design considered patients living with T2D and a rapid renal function decline and addressed a knowledge gap on the potential impact of the modality of physical activity on renal function decline, with no information at the time of the study design and launch. Thus, this study can be considered as a pilot study with an adequate statistical power. It should be noted, however, that no formal power calculation was conducted for the secondary outcomes reported here, including physical activity and sedentary. The trial was specifically powered to detect a clinically meaningful difference in eGFR slope between groups, and the achieved sample size may have been insufficient to detect small-to-moderate effects on behavioral outcomes, particularly given the high variability in physical activity within this population and the multiple measurement time points involved.

The low adherence to the PA objective in the context of the ACTIDIANE study highlights the inherent challenges of conducting long-term interventions in real-life settings with vulnerable populations. Finally, the generalizability of our findings is limited by the specific characteristics of our research population, that is patients living with T2D and showing a rapid decline in renal function. Since pathophysiological studies have clearly identified a relationship between renal function and muscle energy metabolism, the effect of a long-term HIPA program in people living with T2D and an unaltered kidney function remains to be established. Caution is therefore required before generalizing to the whole population of people living with diabetes. More importantly, the generalizability of our findings is limited by the context in which the trial was conducted. A substantial portion of the ACTIDIANE intervention took place during the COVID-19 pandemic, which introduced numerous constraints that were likely to influence both participation and physical activity/sedentarity behaviours. Although adjustments such as e-coaching and t-coaching were implemented with the help of non-academic stakeholders, they could not fully mitigate the effects of social isolation and restricted mobility. Future trials should seek to replicate and refine this type of long-term intervention in more stable contexts and through fully participatory designs, in order to maximize feasibility, engagement, and effectiveness in similar high-risk populations.

## 5 Conclusion

The main findings of the ACTIDIANE trial can be summarized on three key points. Firstly, the difficulty of implementing a structured physical activity program, beyond the question of financial access. The relatively low adherence of the ACTIDIANE HIPA program must lead to research in social sciences to improve commitment and long-term adherence to such programs.

Secondly, despite the non-optimal adherence to the program, the HIPA intervention was associated with an increase in estimated $\dot{V}O_{2}\mathrm{peak}$, which might translate to further health benefits such as life-span and cardiovascular health. This positive effect needs further replication in future RCTs to assess its potential superiority over standard physical activity recommendations.

Thirdly, the HIPA group evidenced no change in sedentary time, showing that the participation of the program did not result in a decrease in other spontaneous moderate or vigorous activities. Increasing the number of observations could help deeply examine whether it ultimately translates to a beneficial decrease in sedentary time and/or increase in MVPA.

The ACTIDIANE is thus a key trial in the context of physical activity in established T2D and associated complications, in a period where the potential of long-term HIPA still remains to be firmly established.

## Data Availability

The raw data supporting the conclusions of this article will be made available by the authors, without undue reservation.

## Appendix A

### Inclusion criteria

- Female or male
- Age: 45 and higher
- Type 2 diabetes with diabetes typing according to widely-accepted clinical and biological criteria
- Subject able to practice physical activity
- With at least 3 available creatinine measurements in the 1 to 5 preceding years showing a rapid renal function decline defined as an eGFR slope below −5 mL/min/yr
- Estimated GFR equal to or higher than 30 mL/min/1.73 m^2^, defined by the CKD-EPI formula, at inclusion visit
- Female participants of child-bearing potential should be willing to make sure to use effective contraception during the study
- Under recommended stable treatment for anti-hypertensive, hypolipidemic and anti-diabetic drugs for 2 months at least
- Ability to give written informed consent after adequate information
- Affiliation with French social security system (in France)

### Non-Inclusion criteria

- Age strictly lower than 45 years
- Indication for cardiovascular rehabilitation (notably patient with ischemic heart disease or coronary revascularisation)
- Treatment with NSAIDs or corticosteroids
- Lower limb amputation (above trans-metacarpal)
- Active proliferative retinopathy (risk of bleeding in case of effort)
- Contre-indication for the participation to physical activity:
  - Severe non-operated valvulopathy
  - Uncontrolled hypertension >180/110 mmHg
  - Thrombus in the left ventricular cavity
  - Unstable coronaropathy, according to physician
- Any condition that would jeopardize patient's safety or would affect the conduct of the study
- Pregnant or breast-feeding women or women of child-bearing potential without effective contraception during the study
- Any situation associated with unreliable cystatin-C determinations, according to patient medical history: HIV positivity, melanoma and thyroid dysfunction
- Simultaneous participation to another research study
- Patients not registered to the social security
- Protected adults (under guardianship and trusteeship)
- Subject unable to express their consent (due to intellectual/mental incapacity)

## Appendix B

Key specifications for selected training centres included:

- To have at least one graduated supervisor able to follow the training proposed by the sports committee of the trial.
- The comply with the regulation on the reception of public, including the approvals concerning hygiene, safety and insurance.
- To have a blood pressure monitor, a blood glucose meter (with the associated consumables) and an automated external defibrillator.
- To have certified equipments (European conformity standard) and a maintenance contract.
- To have a clearly identified care procedure if a participant faced a health problem during an exercise session, in relation with the emergency service of the closest hospital.

An agreement was signed between the training centre and the sponsor of the trial when all conditions were gathered. This agreement was a pre-requisite before the welcome of the first participant.
